# Green Tea Mitigates the Hallmarks of Aging and Age-Related Multisystem Deterioration

**DOI:** 10.14336/AD.2025.0398

**Published:** 2025-04-04

**Authors:** Yusuf Yilmaz

**Affiliations:** Department of Gastroenterology, School of Medicine, Recep Tayyip Erdoğan University, Rize, Türkiye.

**Keywords:** green tea, aging hallmarks, epigallocatechin-3-gallate, healthspan extension, geroscience

## Abstract

Aging is characterized by progressive multisystem deterioration driven by molecular and cellular mechanisms encapsulated in the twelve hallmarks of aging. Green tea (GT), derived from *Camellia sinensis*, has garnered significant scientific interest due to its rich polyphenolic composition, particularly epigallocatechin-3-gallate, and its pleiotropic health benefits. In this narrative review, we explored the multifaceted mechanisms through which GT may mitigate the aging hallmarks. Evidence from *in vitro*, animal, and human studies has shown that GT polyphenols can enhance DNA repair pathways, preserve telomere length, modulate epigenetic aging markers, improve proteostasis and autophagic flux, regulate nutrient-sensing networks, and rejuvenate mitochondrial function. Additionally, GT exhibits anti-inflammatory properties and may restore a physiological gut microbiota composition. Beyond molecular and cellular effects, GT consumption in humans has been associated with improved cognitive function, cardiovascular health, muscle preservation, and metabolic regulation in aging populations. Collectively, these findings highlight GT’s potential as a naturally occurring geroscience intervention capable of addressing the interconnected network of aging processes more comprehensively than single-target pharmaceuticals. Future research should focus on optimizing dosing regimens, exploring synergies with other anti-aging strategies, and investigating personalized responses to GT interventions.

## 1. Introduction

Global life expectancy has increased substantially over the past century, rising from 47.3 years in 1900 to 78.7 years in the United States by 2010 [1]. While this remarkable demographic transition reflects major socioeconomic developments and continuing advancements in medicine, it also poses significant challenges for global healthcare systems in supporting the independence of the elderly population [2]. Accordingly, recent research has documented a widening healthspan-lifespan gap - i.e., the number of years lived with disease or disability - with a global mean of 9.6 years across 183 World Health Organization member states, a disparity that has expanded over the past two decades [3]. This epidemiological trend is particularly concerning as it suggests that increasing lifespan is not entirely matched by parallel improvements in healthspan, ultimately underscoring a growing global burden of chronic age-related diseases and emphasizing the need for innovative strategies to address the complex demands of aging demographics [4].

At the core of the challenges posed by the longevity revolution lies the biological process of aging - characterized by a progressive, multisystem functional decline driven by molecular and cellular mechanisms encapsulated in the twelve traditional hallmarks of aging [5, 6]. These interconnected processes - comprising genome instability, telomere attrition, epigenetic changes, loss of proteostasis, impaired macroautophagy, deregulated nutrient sensing, mitochondrial dysfunction, cellular senescence, stem cell exhaustion, altered intercellular communication, chronic inflammation, and dysbiosis - collectively propel organismal aging and manifest clinically as measurable physiological deteriorations [5, 6]. Cardiorespiratory fitness, for instance, declines linearly by approximately 10% every ten years starting from the fourth decade of life [7]. Similarly, skeletal muscle mass diminishes by 3-8% each decade after 30 years of age, with an accelerated rate of loss after reaching 60 [8]. Collectively, these age-related impairments culminate in an increased vulnerability to stressors, exemplified by frailty syndrome - a condition strongly associated with unfavorable clinical outcomes and increased care dependency [9, 10]. Despite aging’s inevitability, emerging evidence suggests that its trajectory may exhibit a certain degree of plasticity [11], offering potential opportunities to mitigate age-related functional decline through emerging geroscience interventions. However, strategies targeting individual hallmarks of aging continue to encounter substantial translational challenges [12]. For example, while rapamycin and its derivatives have shown promise in bolstering immune, cardiovascular, and integumentary health, they have not exhibited substantial benefits in ameliorating age-related declines in endocrine, muscular, or neurological functions [13]. Similarly, senolytics exhibit heterogeneous effects depending on specific cell types and senescence triggers [14]. In addition, NAD+ boosting strategies - while promising in preclinical models - are constrained by limited knowledge of their pharmacokinetics and pharmacodynamics, particularly regarding bioavailability, metabolism, and target tissue specificity [15]. This impasse in anti-aging medicine has revitalized interest in pleiotropic natural compounds that can simultaneously and positively affect multiple aging hallmarks [16, 17]. This strategy also presents the opportunity for synergistic effects, potentially surmounting the constraints of single-target interventions by tackling the intricate and multidimensional aspects of organismal senescence [18].

As one of the most widely consumed beverages worldwide [19], *Camellia sinensis* derivatives - notably green tea (GT) - have garnered significant scientific interest owing to their distinctive phytochemical composition and pleiotropic health benefits [20-22]. Unlike fermented tea varieties (i.e., black, red, or oolong tea), GT undergoes immediate steaming or pan-firing after harvesting, preserving its high concentration of bioactive polyphenols [23]. This critical step inactivates the polyphenol oxidase enzyme, ultimately halting oxidative degradation and retaining approximately 30-50% of the dry leaf mass as polyphenols [24]. Among biologically active compounds identified in GT, epigallocatechin-3-gallate (EGCG), comprising 50-80% of the total catechin profile, is particularly noteworthy due to its wide range of biological effects [25]. The preservation of EGCG and other polyphenols during processing not only characterizes GT’s chemical signature but also explains its association with various health-promoting properties observed in both preclinical and clinical research [26] - including its antimicrobial, antiviral, anti-inflammatory, antioxidative, neuro-protective, and cardiometabolic regulatory effects [25, 26]. Despite this promising body of evidence, the comprehensive influence of GT on the twelve established hallmarks of aging has not yet been comprehensively discussed. In this narrative review, we sought to address this knowledge gap by examining the multifaceted mechanisms through which GT may attenuate and/or delay senescence, as viewed through the framework of the aging hallmarks. We also examined the potential of GT to influence multiple physiological systems that are vital for supporting healthy aging processes in humans - with a specific emphasis on its impacts on brain, cardiovascular, muscle, body composition, and metabolic functions.

## 2. Methods

We conducted a literature search across five electronic databases (Medline/PubMed, Embase, Scopus, Google Scholar, and ScienceDirect) to identify relevant studies on the effects of GT and its constituents on the hallmarks of aging and age-related functional phenotypes - particularly in the context of cardiorespiratory fitness, Musculo-skeletal function, and frailty. The papers published in English between January 1, 2013 and March 19, 2025, were identified using a strategy that combined the following keywords and Boolean operators: (“green tea” OR “*Camellia sinensis*” OR “epigallocatechin-3-gallate” OR “EGCG”) AND (“aging” OR “senescence” OR “longevity” OR “lifespan”) AND (“hallmarks of aging” OR “geroscience” OR “frailty” OR “functional decline”) (Table 1). The initial search yielded 417 articles, which underwent a meticulous screening process based on their titles and abstracts. Full-text articles were retrieved for review, and reference lists of selected studies were manually searched for additional relevant publications. Priority was given to *in vitro* investigations, animal studies, and human clinical trials that specifically examined GT or its bioactive components in the context of aging processes or age-related conditions. Articles that did not explore this relationship were excluded, as were those published in languages other than English. Data synthesis was conducted using a narrative framework, which categorized the evidence into two main themes: (1) effects of GT on mitigating the twelve hallmarks of aging, and (2) effectiveness of GT in countering age-related deterioration in brain, cardiovascular, muscle, and metabolic functions.

**Table 1:** Literature search strategy.

Criteria	Details
**Databases searched**	Medline/PubMed, Embase, Scopus, Google Scholar, ScienceDirect
**Search focus**	Studies on the effects of green tea and its constituents on the hallmarks of aging and age-related functional decline
**Search period**	From January 1, 2013, to March 19, 2025
**Language restriction**	English
**Search keywords**	(“green tea” OR “Camellia sinensis” OR “epigallocatechin-3-gallate” OR “EGCG”) AND (“aging” OR “senescence” OR “longevity” OR “lifespan”) AND (“hallmarks of aging” OR “geroscience” OR “frailty” OR “functional decline”)
**Inclusion criteria**	Articles specifically addressing green tea or its constituents in relation to aging processes
**Exclusion criteria**	Articles not focused on the relationship between green tea and aging or age-related conditions; non-English articles

## 3. Effects of GT on mitigating the twelve hallmarks of aging

## 3.1. Green tea and genome instability

The accumulation of age-related DNA damage is influenced by a multitude of intrinsic and extrinsic factors - including the decline of endogenous DNA repair mechanisms, errors during DNA replication, exposure to chemical and physical mutagens, and oxidative stress [26]. Consequently, genome instability has emerged as a pivotal factor in the aging process, as the increasing mutational burden adversely impacts cell and tissue homeostasis by altering gene expression profiles or triggering cellular senescence or apoptosis [27]. Mounting evidence indicates that GT polyphenols have the capacity to mitigate genome instability through complementary DNA-protective pathways operating at multiple molecular levels. *In vitro* data obtained from the yeast *Saccharomyces cerevisiae* have shown that GT extract can promote distinct DNA repair pathways - particularly nucleotide excision repair (NER) and homologous recombination - in response to ultraviolet (UV)-induced DNA damage [29]. Accordingly, pretreatment of UVB-irradiated yeasts with GT effectively increased the expression of key DNA repair genes (e.g., *RAD4, RAD14, RFA1, RAD51*, and *RAD52*), ultimately preserving genome stability, reducing mutation frequency, and improving survival rates of irradiated cells. Interestingly, the total GTE extract demonstrated superior DNA-protective effects compared to individual bioactive components like EGCG or caffeine [29]. In a separate study conducted on cultured neuronal cells, tea polyphenols were found to attenuate methamphetamine-induced cell injury by suppressing oxidative DNA damage through an increased expression of DNA repair-associated proteins, including p-ATM and p-Chk2 [30]. *In vivo* studies corroborated the genome-protecting effects of GT. Accordingly, Katiyar et al. [31] demonstrated that GT polyphenols in drinking water (0.2%, w/v) accelerated the repair of UV-induced DNA damage, evidenced by a 59% reduction in cyclobutane pyrimidine dimer-positive (CPD+) cells in the skin (P < 0.001) and a two-fold decrease in CPD+ cell migration to draining lymph nodes. Interestingly, the genoprotective effects of GT were contingent upon functional NER mechanisms, as demonstrated by their inefficacy in NER-deficient mice but significant prevention of UV-induced immune-suppression in NER-proficient mice [31]. Human clinical studies have further validated the genome-stabilizing effects of GT. Accordingly, a randomized, placebo-controlled crossover trial demonstrated that both acute (single dose of 200 mL, 1.5% w/v) and regular (200 mL, 1% w/v twice daily for 7 days) GT consumption significantly reduced lymphocytic oxidative DNA damage by approximately 30% compared to water control [31]. This genoprotective effect was accompanied by a significant increase in the activity of human oxoguanine glycosylase 1 (hOGG1), an enzyme critical for repairing oxidative DNA base lesions [32]. Elevated hOGG1 activity in lymphocytes was observed shortly after acute GT ingestion (60-120 min post-dose) and following sustained intake over 7 days, suggesting that GT’s effects involve both immediate post-translational modifications and longer-term adaptive responses in DNA repair capacity [31]. Furthermore, GT consumption significantly increased expression of heme oxygenase-1, suggesting that the activation of redox-sensitive cytoprotective pathways likely contributes to an improved genome stability [31]. Another randomized controlled trial conducted in heavy smokers demonstrated that consuming four cups (960 mL) of decaffeinated GT per day significantly reduced urinary 8-hydroxyde-oxyguanosine - a biomarker of oxidative DNA damage - compared to water control [33]. This protective effect was particularly pronounced in genetically defined subpopulations with specific detoxification capacities [33]. A separate randomized, placebo-controlled crossover trial involving 18 healthy volunteers demonstrated that consuming 300 mL of 1% GT daily for 4 weeks significantly enhanced lymphocytic DNA resistance to oxidative challenge and reduced basal oxidative DNA damage by approximately 20% compared to water control [34]. Similarly, in patients with type 2 diabetes - a condition known to be associated with accelerated aging [35] - GT consumption was associated with approximately 15% lower DNA damage and 50% higher hOGG1 activity compared water control [36]. The consistent results across *in vitro*, animal, and human studies collectively indicate that regular GT consumption may represent an effective and accessible strategy for mitigating aging-associated genome instability and maintaining genomic integrity by enhancing DNA repair pathways and reducing oxidative DNA damage.

## 3.2. Green tea and telomere attrition

Telomeres - comprising tandem TTAGGG nucleotide repeats - form protective caps at chromosome termini that are essential for maintaining chromosomal stability. These structures extend from several to tens of kilobases and terminate in a 3’ single-stranded guanine-rich overhang spanning 75-300 nucleotides [37]. In addition, the telomeric region is protected by the shelterin complex, a specialized assembly of six proteins that plays a crucial role in preserving telomere homeostasis and preventing aberrant DNA damage responses at chromosome ends [38]. With each cell division, telomeres progressively shorten due to the end-replication problem, eventually reaching a critical length that activates DNA damage signaling pathways, leading to replicative senescence and permanent cell cycle arrest [39]. As a result, telomere dysfunction is closely associated with cellular aging, and shortened telomeres have been consistently observed in numerous age-related disorders [40]. Notably, GT derivatives have been suggested to exert antiaging effects via prevention of telomere shortening. Accordingly, *in vitro* experiments in cardiomyocytes have demonstrated that EGCG can effectively prevent hydrogen peroxide-induced telomere attrition, ultimately inhibiting the telomere-dependent apoptotic pathway [41]. In an animal study, Oyama et al. [42] examined how EGCG can affect cardiac remodeling and telomere biology in heart/muscle-specific MnSOD-deficient mice - which are prone to develop congestive heart failure and dilated cardiomyopathy. Results showed that EGCG administration significantly improved survival rates and prevented cardiac dilatation and contractility reduction - while preserving telomere length and telomerase activity that were compromised in untreated knockout mice. Similarly, human clinical investigations have revealed that regular GT consumption is associated with reduced telomere attrition. In a study conducted in 2,006 elderly Chinese individuals, higher tea consumption was significantly associated with longer telomeres in male participants, with those drinking more than three cups daily having telomeres approximately 0.46 kb longer than those consuming less than 0.28 cups daily, equivalent to about 5 years of life difference [43]. Recently, Sohn et al. [44] investigated the relationship between beverage consumption (GT *versus* soft drinks *versus* coffee) and changes in leukocyte telomere length over a 6-year period in 1,952 subjects. Daily consumption of at least one cup of GT was associated with less telomere shortening compared to non-consumers, with this protective effect being more pronounced in women and younger participants (50-64 years). Conversely, soft drink consumption was linked to accelerated telomere shortening in women - potentially accelerating biological aging - while coffee did not show a significant impact on telomere dynamics [44]. In a cross-sectional interventional study, Nonino et al. [45] examined the effect of decaffeinated GT supplementation on telomere length in obese women, which were compared to normal-weight women. At baseline, obese women had significantly shorter telomeres than normal-weight individuals, with an inverse relationship being evident between telomere length and body mass index even after adjustment for age. Notably, after eight weeks of GT supplementation, a marked 103% increase in telomere length was observed in obese participants [45]. Intriguingly, there is evidence to suggest that GT may influence telomere dynamics in a context-dependent manner. Specifically, while GT derivatives have been shown to mitigate telomere attrition linked to cellular senescence and age-related pathologies, they can paradoxically promote telomeric shortening in neoplastic cells. Accordingly, *in vitro* experiments demonstrated that prolonged exposure of U251 glioblastoma cells to sublethal concentrations of EGCG progressively compromises telomere integrity, ultimately precipitating cellular senescence following 98 days of treatment [46]. Concordantly, incubation of human small-cell lung carcinoma cells with EGCG inhibited telomerase activity by 50-60% within 24 hours, as determined by quantitative PCR-based telomeric repeat amplification protocol [47].

## 3.3. Green tea and epigenetics alterations

Epigenetic modifications - including altered DNA methylation patterns (characterized by global hypomethylation alongside CpG island hyper-methylation) [49], chromatin structure remodeling (comprising heterochromatin loss and disrupted 3D genome organization) [50], histone modifications [51], and changes in non-coding RNA (ncRNA) profiles [52] - are among the most conserved hallmarks of aging [48]. Accordingly, age-associated epigenetic modifications have been linked to dysregulated transcriptional programs that contribute to cellular senescence and genomic instability [48]. In terms of DNA methylation, a recent 18-month randomized controlled trial demonstrated that daily GT consumption (3-4 cups per day) as part of a Green-Mediterranean (Green-MED) diet was significantly associated with a reduction in methylation age, even after adjusting for age, sex, baseline methylation age, and weight loss. The Green-MED diet, an enhanced version of the traditional Mediterranean diet, was calorie-restricted (1500-1800 kcal/day for men and 1200-1400 kcal/day for women) and prioritized plant-based and polyphenol-rich foods while limiting processed and red meat. Key components of this diet included 28 g/day of walnuts, 3-4 cups/day of GT, and 500 mL/day of Mankai (a *Wolffia globosa* duckweed cultivar) - which collectively contributed an extra 800 mg/day of polyphenols. Notably, participants adhering to the Green-MED diet exhibited an average favorable difference of approximately 8.9 months between their observed and expected epigenetic age [53]. *In vitro* experiments have also shown that EGCG can promote chromatin relaxation in human endothelial cells by acting as a histone deacetylase inhibitor in both cellular and cell-free models, while simultaneously increasing histone acetylation and reducing the expression of heterochromatin binding proteins [54]. In mice, long-term administration of EGCG prevented aging-related cardiac diastolic dysfunction through significant histone modifications in heart tissue [55]. Accordingly, EGCG treatment inhibited both the expression and activity of histone deacetylase 1 while reducing its binding near the cardiac troponin I (cTnI) promoter region, ultimately counteracting the age-associated decline in cTnI expression [55, 56]. Emerging evidence also suggests that tea constituents may play a role in mitigating epigenetic aging processes by modulating ncRNA expression. Accordingly, Kim et al. [57] demonstrated that nc886, a long non-coding RNA typically downregulated during fibroblast replicative senescence, can be significantly upregulated by tea-derived preparation. Collectively, these findings indicate that GT polyphenols have significant potential for epigenetic reprogramming, which can effectively attenuate specific aspects of aging and age-related diseases.

## 3.4. Green tea and loss of proteostasis

As organisms age, the proteostasis network - a sophisticated system consisting of molecular chaperones, proteolytic machinery, and regulatory components - experiences progressive functional decline [58]. This deterioration disrupts proteome homeostasis, a vital process governing protein synthesis, folding, structural integrity, and degradation. The resulting loss of proteostasis is marked by the accumulation of oxidized (carbonylated), misfolded, and aggregated proteins, which are widely recognized as hallmarks of cellular aging [59]. Emerging evidence suggests that EGCG holds promise in regulating proteostasis by addressing protein misfolding and aggregation. Specifically, EGCG has been shown to inhibit fibrillization and facilitate the disassembly of misfolded aggregated proteins, potentially mitigating the risk of age-related neurodegenerative diseases [60]. Furthermore, EGCG can reduce amyloid cytotoxicity and transform existing fibrils into non-toxic amorphous species incapable of propagating further aggregation. Intriguingly, oxidized EGCG enhances fibril remodeling through Schiff base formation and crosslinking mechanisms [61]. In addition to its effects on protein aggregation, EGCG has demonstrated the ability to inhibit the chymotrypsin-like activity of the proteasome both *in vitro* and *in vivo* at concentrations achievable through GT consumption [62]. By suppressing proteasomal activity, EGCG may lead to the accumulation of natural proteasome substrates, thereby influencing cellular processes. Animal studies further support the role of GT extract in modulating proteostasis; for instance, it has been shown to block tendon crosslinking in adult mice and reduce fluorescent products associated with collagen aging [63]. These findings suggest an antioxidant mechanism through which GT may favorably impact protein homeostasis.

## 3.5. Green tea and disabled macroautophagy

Disabled macroautophagy - a highly conserved cellular recycling mechanism that targets and eliminates damaged organelles, protein aggregates, and dysfunctional components through lysosome-dependent degradation pathways - represents a fundamental hallmark of aging [64, 65]. This critical quality control process, which allows for the orderly degradation and recycling of cellular constituents, experiences a progressive functional decline with advancing age, with autophagic flux decreasing significantly in senescent cells [66]. Interestingly, human research has shown that healthy centenarians are characterized by constitutively elevated levels of autophagy [67]. GT-derived polyphenols have been found to stimulate autophagy via multiple pathways, including inhibition of mechanistic target of rapamycin (mTOR) signaling in response to endoplasmic reticulum stress, as well as through AMP-activated protein kinase (AMPK) [68] and sirtuins [69]. Interestingly, long-term consumption of EGCG in aged mice has been shown to enhance sirtuin expression and increase the autophagic flux, particularly in adipose and intestinal tissues, ultimately increasing lifespan [70]. In another study focusing on early vascular aging induced by a high-fat diet in Wistar rats, GT polyphenols treatment alleviated arterial wall disorganization by inhibiting cell senescence and promoting autophagy in endothelial cells via sirtuin 3 signaling [71]. A separate animal investigation demonstrated that EGCG primes autophagy to clear cellular damage during muscle disuse (hindlimb suspension) in aged rats, while suppressing excessive autophagy during recovery, suggesting a therapeutic strategy against age-related sarcopenia [72]. Recently, GT polyphenols have been shown to induce mitophagy (i.e., the removal of damaged mitochondria by autophagy) in human fibroblasts at low concentrations (5-10 μM), suggesting their anti-aging properties may at least in part arise from an improved autophagy-mediated mitochondrial health [73]. Based on this evidence, it has been proposed that autophagy stimulation through GT consumption may significantly reduce the incidence and progression of major age-related disorders, including cancer and Alzheimer’s disease (AD) [74, 75].

## 3.6. Green tea and dysregulated nutrient-sensing

Age-associated dysregulation of nutrient-sensing mechanisms manifests through perturbations in evolutionarily conserved signaling cascades, notably involving the insulin-like growth factor 1 (IGF-1), AMPK, mTOR, and sirtuins pathways [76]. These interconnected systems collectively coordinate cellular metabolic homeostasis and energy regulation. The critical role of these molecular signals in aging biology is evidenced by lifespan extension observed in multiple model organisms following dietary restriction, which attenuates activity in nutrient-responsive networks [77]. Notably, GT polyphenols have the potential to modulate nutrient-sensing pathways that become dysregulated during organismal aging. Accordingly, EGCG may mimic the actions of insulin and IGF-1 by promoting the nuclear efflux of the atrophy-associated transcription factor Foxo1 in skeletal muscle fibers - suggesting a positive influence on age-related loss of muscle mass and strength [78]. In addition, GT polyphenols can activate AMPK in adipose tissue and liver, enhancing fatty acid oxidation while suppressing lipid synthesis [79]. This AMPK-mediated modulation has also been found to reduce obesity-related markers, including visceral fat accumulation, insulin resistance, and pro-inflammatory cytokines [79] - all factors associated with an accelerated aging process [80]. Notably, EGCG may also act as an ATP-competitive inhibitor of both phosphoinositide 3-kinase (PI3K) and mTOR [81] - two mechanisms that mimic systemic nutrient deprivation signals [82, 83] known to promote longevity [77]. Importantly, mTOR inhibition has been shown to extend mammalian lifespan by attenuating nutrient-sensing pathways [84]. Furthermore, GT extract can restore cardiomyocyte contractility in diabetic models by enhancing mitochondrial metabolic activity and calcium handling dynamics through sirtuin 1 upregulation [85]. Consequently, by simultaneously modulating multiple nutrient-sensing pathways, GT derivatives may help overcome the limitations of single-targeted anti-aging interventions - which often exhibit limited efficacy due to compensatory crosstalk among the PI3K, mTOR, AMPK, and sirtuins regulatory nodes.

## 3.7. Green tea and mitochondrial dysfunction

Mitochondrial dysfunction - characterized by accumulating mitochondrial DNA mutations, reduced ATP synthesis efficiency, compromised membrane potential, and dysregulated quality control mechanisms - represents a central feature of aging that drives cellular senescence across multiple tissues [86]. Notably, EGCG is a well-characterized mitochondria-targeting poly-phenol, which has been shown to regulate oxidative metabolism while counteracting age-related mitochondrial dysfunction [87]. In *Caenorhabditis elegans*, EGCG can extend lifespan through mechanisms involving amplified mitochondrial biogenesis and improved respiratory capacity [88]. In Hepa1-6 hepatoma cells, synthetic EGCG derivatives significantly increased cytochrome c levels (1.4-fold), oxygen consumption rates (2.1-fold), ATP production (1.5-fold), and NAD+/NADH ratios (2.2-fold) - suggesting a consistent stimulation of oxidative phosphorylation [89]. Obese murine models further confirmed EGCG’s capacity to stimulate mitochondrial DNA replication [90], suggesting therapeutic potential for age-related metabolic disorders. Emerging evidence also highlights mitophagy induction as a critical anti-aging mechanism of green tea compounds. Accordingly, catechin-rich extracts (≥ 98% polyphenols) effectively promoted dose-dependent mitophagy activation in human fibroblasts, promoting mitochondrial turnover and homeostasis [73]. Pilot findings also indicate that GT extracts may exert a regulatory influence on mitochondrial function in humans. Specifically, a study by Venables et al. [91] examined the effects of combining GT extract supplementation with cycling exercise in a cohort of 12 healthy, normal-weight males. The results revealed that consuming GT extracts one hour prior to exercise significantly enhanced fat oxidation rates compared to a control group receiving a placebo. This outcome supports the notion that GT can favorably affect not only mitochondrial function but also bioenergetic control - potentially acting as a mitochondrial rejuvenation factor [92].

## 3.8. Green tea and cellular senescence

Cellular senescence represents a multifaceted biological phenomenon characterized by the irreversible cessation of cell division in response to various stressors [93]. A key feature of senescent cells is their ability to develop a bioactive secretory profile - known as senescence-associated secretory phenotype (SASP) - which plays a pivotal role in influencing the tissue microenvironment via paracrine signaling mechanisms [94]. Remarkably, GT-derived compounds, particularly EGCG, have demonstrated significant anti-senescence effects across diverse experimental models. In aging mice, chronic EGCG consumption has been shown to attenuate cellular senescence by mitigating DNA damage, inhibiting cell cycle regulators, and suppressing SASP-associated factors - especially in vulnerable adipose and intestinal tissues [70]. Similarly, EGCG elicited protective effects in preadipocytes subjected to H_2_O_2_-induced premature senescence [95]. In human mesenchymal stem cells, EGCG pre-treatment (50-100 μM) effectively reduced H_2_O_2_-induced senescence by activating nuclear factor erythroid 2-related factor 2 - a key regulator of antioxidant defense pathways [96]. Furthermore, the proportion of senescence-associated β-galactosidase (SA-β-gal)-positive cells was significantly diminished in EGCG-pretreated groups compared to untreated H_2_O_2_-exposed controls [96]. Comparable findings were observed in endothelial cells, where EGCG treatment (100 μM) markedly reduced senescence-associated characteristics, including decreased SA-β-gal activity and reversal of elevated expression of senescence-related genes (e.g., *CDKN1A*, *CDKN2A*, and *CDKN2B*) [97]. Intriguingly, EGCG demonstrated superior anti-senescent efficacy compared to other senolytic and senomorphic agents like quercetin and resveratrol within this experimental framework [97]. In another comparative study examining the effects of bioactive compounds - including EGCG, anthocyanidin, and resveratrol - on 3T3-L1 preadipocytes where senescence was induced by exposure to 5-bromodeoxyuridine, EGCG proved to be the most effective agent in both reducing pro-inflammatory cytokine secretion and *CDKN1A* expression, while simultaneously activating sirtuin 3 [98]. Additionally, at concentrations ranging from 50 to 100 μM, EGCG effectively prevented cellular senescence in rat vascular smooth muscle cells, human dermal fibroblasts, and human articular chondrocytes by suppressing p53 acetylation [99]. This mechanism was further linked to differential nuclear translocation of EGCG in proliferating *versus* senescent fibroblasts, ultimately facilitating cell cycle recovery [99].

## 3.9. Green tea and stem cell exhaustion

Stem cell exhaustion refers to the progressive decline in the regenerative potential of tissue-specific stem cells [100]. This phenomenon is driven by intrinsic and extrinsic factors that disrupt the delicate balance between stem cell quiescence and proliferation - ultimately impairing their ability to migrate from the bone marrow and engraft to damaged tissue to promote regeneration and repair [101]. EGCG has been recently shown to increase the viability of adipose-derived stem cells (ADSCs) under high-glucose stress conditions [102]. Additionally, in an animal model of type 1 diabetes, EGCG-pretreated ADSCs significantly outperformed ordinary ADSCs in addressing islet size reduction and fibrotic collagen deposition [102]. In a separate study, EGCG has been found to attenuate the pro-inflammatory effects of extracellular vesicles (EVs) derived from triple-negative breast cancer (TNBC) cells on human ADSCs [103]. Specifically, EGCG altered the genetic content of TNBC-secreted EVs under hypoxic conditions, ultimately mitigating senescence markers such as p21 and β-galactosidase [103] and inhibiting the activation of proinflammatory nuclear factor (NF)-κB [104]. In irradiated mice, EGCG promoted radioprotection by increasing the number of intestinal stem cells, while reducing DNA damage, apoptosis, and ferroptosis, and activating the Nrf2 signaling pathway to mitigate oxidative stress [105]. Interestingly, another study found that EGCG effectively induced a marked translocation of Nrf2 into cell nuclei and was effective in preventing oxidative stress-induced cellular senescence in human mesenchymal stem cells [96]. In aged rats, GT extract attenuated muscle loss during hindlimb suspension and increased satellite cell proliferation and differentiation in plantaris and soleus muscles during recovery, while decreasing oxidative stress [106]. Notably, epicatechin gallate - a GT-derived polyphenol - has been shown to promote myogenic differentiation through the induction of myogenic markers in satellite and C2C12 myoblast cells [107] - with similar effects having been reported for EGCG [108]. Additionally, in aging rat brains, EGCG co-cultured with ADSCs improved neurogenic activity, tissue integrity, and cell survival through the activation of p-Akt and sirtuins, while boosting antioxidant activity via the Nrf2 and heme oxygenase-1 pathways [109].

## 3.10. Green tea and altered intercellular communication

Aging disrupts intercellular communication through several mechanisms, including alterations in gap junctions, dysregulated EVs trafficking, and impaired circulating microRNA signaling networks [110, 111]. GT derivatives have demonstrated protective effects against reductions in gap junctional intercellular communication (GJIC) and connexin expression under various pathological conditions. Specifically, EGCG has been shown to mitigate the downregulation of Connexin 43 in cardiomyocytes exposed to elevated glucose concentrations [112] and in endothelial cells subjected to serum deprivation [113]. Furthermore, GT administration prevents the decline of GJIC observed in the hepatic tissues of mice exposed to pentachlorophenol - a known carcinogen in mouse liver [114]. Recent findings have also highlighted EGCG’s ability to significantly inhibit EVs secretion from senescent vascular endothelial cells [97]. Interestingly, while EVs derived from senescent cells are capable of amplifying lipopolysaccharide-induced pro-inflammatory responses in monocytes, EVs isolated from EGCG-treated cells markedly attenuated this inflammatory cascade [97]. Notably, EGCG may also positively influence the neuro-immune cross-talk - which is typically disrupted in aging - by enhancing neurotrophins release and supporting intestinal homeostasis via positive modulation of the brain-gut axis [115]. From a clinical standpoint, acute GT supplementation has been reported to reduce the expression of 62 circulating microRNAs involved in critical pathways regulating growth, differentiation, and fibrosis in women with obesity following a high-fat, high-saturated meal consumption [116].

## 3.11. Green tea and chronic inflammation

Chronic inflammation that occurs during the aging process - commonly referred to as inflammaging - is considered central to the pathogenesis of age-related chronic diseases by inducing sustained, macrophage-driven, low-grade immune activation triggered by cell debris [117-119]. Growing evidence indicates that GT-derived compounds may exert potent anti-inflammatory activities through multiple mechanisms of action [120], suggesting their potential usefulness to combat inflammaging. Accordingly, EGCG effectively inhibits the proinflammatory NF-κB signaling pathway by attenuating NF-κB-mediated transcriptional activation through covalent modification [121]. The reduction of age-associated inflammatory response through NF-κB inhibition is also considered a key mechanism by which EGCG may contribute to lifespan extension in healthy rats [122]. In a model exploring systemic inflammation-induced cognitive decline in elderly rats, EGCG proved effective in mitigating the cytokine surge triggered by lipopolysaccharide exposure [123]. Additionally, research into the impact of senescent preadipocyte-secreted factors on age-related immune cell dysfunction revealed that EGCG treatment markedly attenuated proinflammatory responses in aged macrophages [124]. EGCG can also exert neuroprotective effects by inhibiting both canonical and non-canonical inflammasome activation via the toll-like receptor (TLR) 4/NF-κB signaling pathway [125]. These findings, derived from *in vitro* studies on lipopolysaccharide- and amyloid β-induced microglial dysfunction, as well as *in vivo* investigations in APP/PS1 transgenic mice [125], underscore the potential of EGCG in alleviating neuroinflammation-associated cognitive decline linked to aging [126, 127]. In line with this possibility, tea polyphenols may also effectively suppress the activation of the brain TLR4/NF-κB inflammatory signaling pathway triggered by intestinal dysbiosis [128]. In male Kunming mice, GT polyphenols have shown remarkable efficacy against with D-galactose-induced liver aging by suppressing the increased expression of pro-inflammatory cytokines in the hepatic parenchyma [129]. In human studies, anti-inflammatory mechanisms - driven by polyphenols - have also been advocated to explain the potential of regular tea consumption as a modifiable lifestyle factor to attenuate biological aging [130] and age-related diseases - including atherosclerotic vascular disorders, metabolic syndrome, and type 2 diabetes [131].

## 3.12. Green tea and dysbiosis

During aging, the gut microbiota exhibits a notable reduction in bacterial diversity, taxonomic shifts, and a decreased abundance of beneficial microbial signatures and species [132]. With respect to the two dominant bacterial phyla, the relative abundance of Firmicutes tends to increase with aging, while that of Bacteroidetes decrease from childhood to elderly age [133]. Other age-related alterations in microbiota composition include a decreased abundance of *Faecalibacterium prausnitzii*, *Clostridium* cluster XIVa, and members of the Actinobacteria phylum [132]. Furthermore, several metabolically significant species - including *Ruminococcus bromii* [134], *Akkermansia muciniphila* [135], and *Ruminococcus gnavus* [136] - are underrepresented in the gut microbiota with advancing age. GT has been shown to interact with intestinal microbial communities in a complex, bidirectional manner [137]. While on the one hand GT can promote the growth of beneficial bacterial species while reducing colonization by pathogens, on the other hand gut bacteria can actively metabolize GT compounds [138] - potentially modulating their biological activities [139]. Notably, GT has been shown to counteract the primary age-associated gut microbiota signatures. Specifically, GT ingestion has been associated with an increased Firmicutes-to-Bacteroidetes ratio [140] and higher relative abundance of the main beneficial species that tend to decrease with age - including *Faecalibacterium prausnitzii* [141], *Clostridium* cluster XIVa [142], Actinobacteria [143], and *Akkermansia muciniphila* [144].

## 4. Effectiveness of GT in countering age-related multisystem deterioration in humans

**Figure 1.**
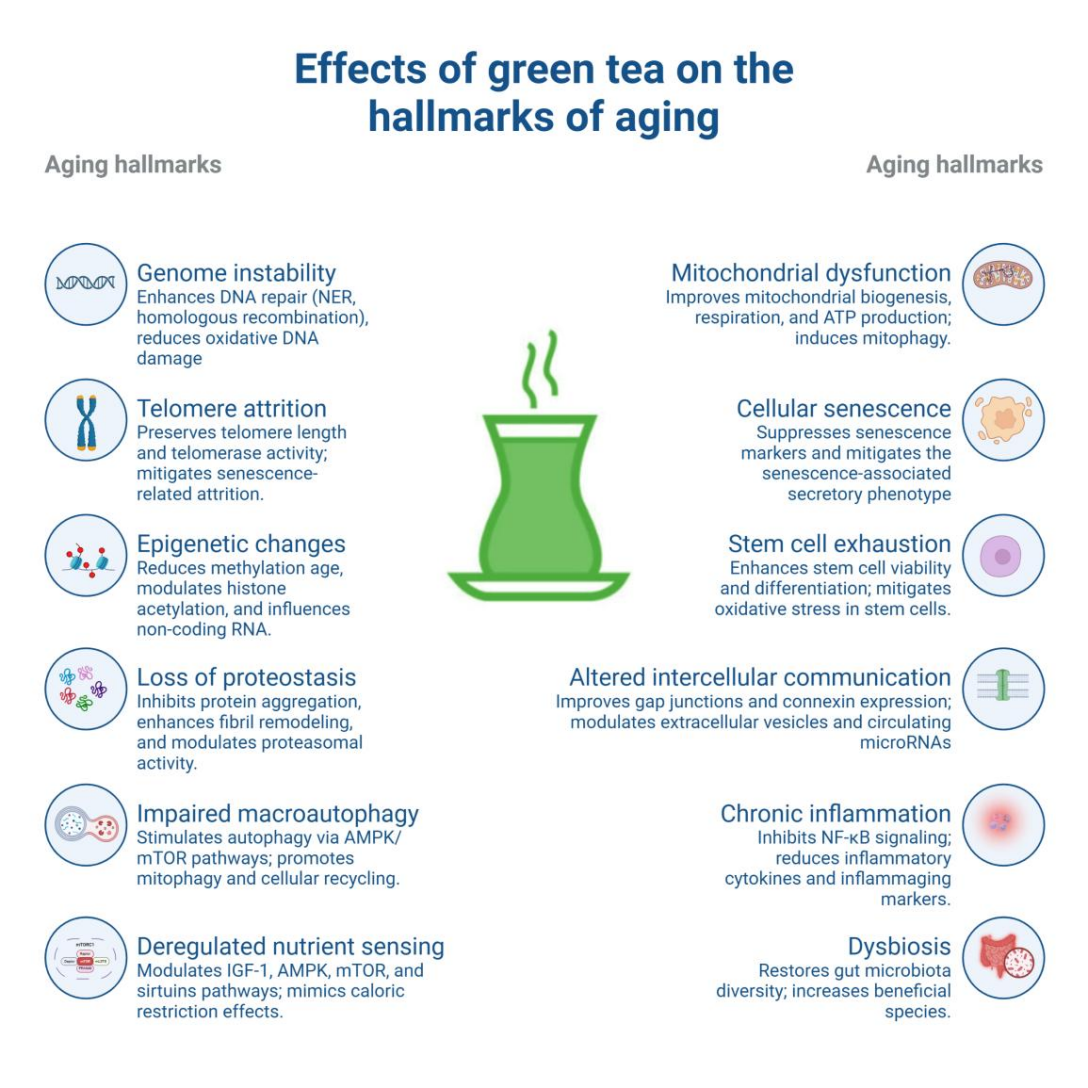
Impact of green tea on the twelve hallmarks of aging (*Created with* Biorender.com).

The evidence concerning GT’s impact on the twelve hallmarks of aging highlights its capacity to positively modulate, at least in part, the core elements of the senescence process (Fig. 1). Such influence is pivotal for extending healthspan, promoting resilience, and reducing the likelihood of frailty in the elderly population. We will now examine the available human and clinical data that supports GT’s effectiveness in mitigating age-related multisystem decline across an array of organs and systems - including brain function, cardiovascular health, muscle function, and metabolic regulation.

## 4.1. Green tea and brain function

The trajectory of cognitive capabilities serves as a fundamental determinant in shaping both the quality of life and subjective well-being experienced by individuals as they navigate the transition from middle adulthood into old age [145]. A recent longitudinal study investigated 1,155 middle-aged individuals (aged 44-66 years at baseline in 1995) to explore the relationship between specific GT consumption patterns assessed in 1995 and 2000 and long-term cognitive outcomes, as determined by neuropsychological assessments conducted about two decades later (2015-2025) [146]. The analysis revealed that moderate GT consumption, specifically 2-3 cups per day, was associated with a significant 44% reduction in the risk of cognitive decline after adjusting for potential confounding variables. Notably, this protective relationship did not extend to higher consumption levels of four or more cups per day. Sex-stratified analysis showed a particularly pronounced protective effect among male participants, with a 62% lower risk. These findings suggest that incorporating moderate amounts of green tea during midlife may provide neuroprotective benefits against cognitive decline, potentially with stronger effects in men [146]. A separate double-blind, randomized, controlled trial conducted in Japan similarly reported that regular consumption of decaffeinated GT catechins (336.4 mg daily) may enhance working memory capabilities, providing potential cognitive benefits for adults aged 50-69 years [147]. Notably, a single dose of GT catechins resulted in a significant reduction in incorrect responses during the Continuous Performance Test, indicating an immediate improvement in attentional control. In addition, following 12 weeks of daily GT catechins intake, participants demonstrated a faster response time in the two-back component of the test [147]. Importantly, several systematic reviews and meta-analyses have demonstrated that tea intake consumption may help reduce the burden of dementia [148-151], a growing public health concern among the elderly population. A noteworthy recent meta-analysis by Jang et al. [151] reported that regular tea consumption correlates with a 29% lower risk of developing all-cause dementia. When examining specific dementia subtypes, the researchers found that tea drinking was associated with a 12% reduction in AD risk and a more pronounced 25% decrease in vascular dementia risk. The protective benefits of tea appeared more substantial in populations characterized by sedentary lifestyle, advanced age, APOE ε4 carriers, and individuals with smoking habits [151].

## 4.2. Green tea and cardiovascular function

Age-related physiological changes significantly impact the cardiovascular system through multiple interconnected mechanisms - including hypertension, altered local regional blood flow, endothelial dysfunction, and atherosclerotic vascular disease. A meta-analysis examining the effects of GT on blood pressure included 24 clinical trials with a total of 1,697 participants. The aggregated findings demonstrated that GT consumption was associated with a modest but statistically significant reduction in both systolic blood pressure (SBP decreased by 1.17 mm Hg) and diastolic blood pressure (DBP decreased by 1.24 mm Hg) [154]. Another recent meta-analysis of nine studies in 680 healthy individuals reported that GT supplementation yielded a reduction in SBP by 2.99 mm Hg and a decrease in DBP by 0.95 mm Hg [155]. Regarding age-related regional blood flow alterations, a recent randomized, single-blind, placebo-controlled crossover study evaluated GT extract (~500 mg EGCG) *versus* placebo with an oral nutritional supplement in twelve healthy older adults. Using ultrasound and blood sampling techniques, the authors found that GTE significantly increased vastus lateralis microvascular blood volume at 3 and 4 h post-supplementation - suggesting an improved leg muscle perfusion [156]. With respect to endothelial dysfunction, a systematic review of randomized controlled trials revealed that consuming an average daily dose of 500 mL of tea (~ two cups) led to an improvement in flow-mediated dilation, increasing arterial diameter by approximately 2.6% compared to a placebo [157]. Interestingly, this beneficial vascular effect appears to be independent of EGCG, suggesting other bioactive compounds in GT may be responsible [158]. Another meta-analysis that included 772,922 participants suggested that regular GT consumption, particularly at low-to-moderate levels (1-4 cups daily), may confer protection against coronary heart disease [159]. In a similar study on 259,267 subjects, a dose-dependent relationship between GT consumption and cardiovascular health was observed, with non-consumers showing significantly higher risks of cardiovascular disease, intracerebral hemorrhage, and cerebral infarction compared to light consumers [160]. Notably, moderate green tea intake (1-3 cups daily) was associated with reduced risks of myocardial infarction and stroke, while higher consumption levels (≥ 4 cups daily) demonstrated even greater protective effects against myocardial infarction [160].

## 4.3. Green tea and muscle function

Sarcopenia refers to the progressive skeletal muscle deterioration affecting mass, strength, and function in older adults, which lowers quality of life, impairs mobility, and increases the risk of serious injuries and adverse clinical outcomes [161]. This condition affects approximately 10-16% of the elderly population worldwide and is associated with substantially increased healthcare utilization [162]. Data from the United States have shown that the total estimated hospitalization cost for individuals with sarcopenia reached 40.4 billion dollars [163] - suggesting the critical importance of improving muscle function and combating sarcopenia as essential components of healthy aging strategies. A recent cross-sectional study revealed a robust inverse association between habitual GT consumption and sarcopenia prevalence among community-dwelling Japanese older adults [164]. Accordingly, participants consuming ≥1 cup/day of GT demonstrated a striking 68.8% reduction in sarcopenia risk compared to those consuming < 1 cup/week after adjustment for potential confounding factors [164]. In a pilot randomized controlled trial, Tokuda and Mori [165] investigated the effects of combining tea catechins (TCs) and essential amino acids (EAAs) supplementation with resistance exercise (RE) on skeletal muscle mass (SMM) in older adults with sarcopenia. The results revealed significantly higher SMM in the RE + EAAs + TCs group compared to the RE-only group - suggesting that supplementing with TCs and EAAs may boost the positive effects of RE on SMM in the elderly.

## 4.4. Green tea and metabolism

The respiratory quotient - which reflects the balance between carbohydrate and fat oxidation by measuring the ratio of carbon dioxide exhaled to oxygen consumed - exhibits an age-related decrease that parallels a decline in lipid oxidation [166]. This reduction can be effectively counteracted by supplementation with GT catechins [167], thereby supporting metabolic health during aging. Another critical metabolic factor for healthy aging involves glucose regulation [168], as shown by a significantly higher insulin sensitivity in human centenarians compared to subjects aged over 75 years [169]. Notably, in a meta-analysis comprising 17 trials and involving 1133 participants, GT consumption significantly decreased fasting glucose levels by -0.09 mmol/L while reducing fasting insulin levels by -1.16 μIU/mL [170] - ultimately highlighting its potential benefits for glucose metabolism. Evidence also suggests that liver mass declines by 20% to 40% in older adults [171], who are also at an increased risk of developing steatotic liver disease (SLD) [172]. Interestingly, a meta-analysis of four studies demonstrated that GT supplementation may mitigate SLD, as evidenced by significant reductions in liver enzymes - including alanine aminotransferase by -12.81 U/L and aspartate aminotransferase by -10.91 U/L - along with a reduction in body mass index and improvements in lipid profiles [173].

**Figure 2.**
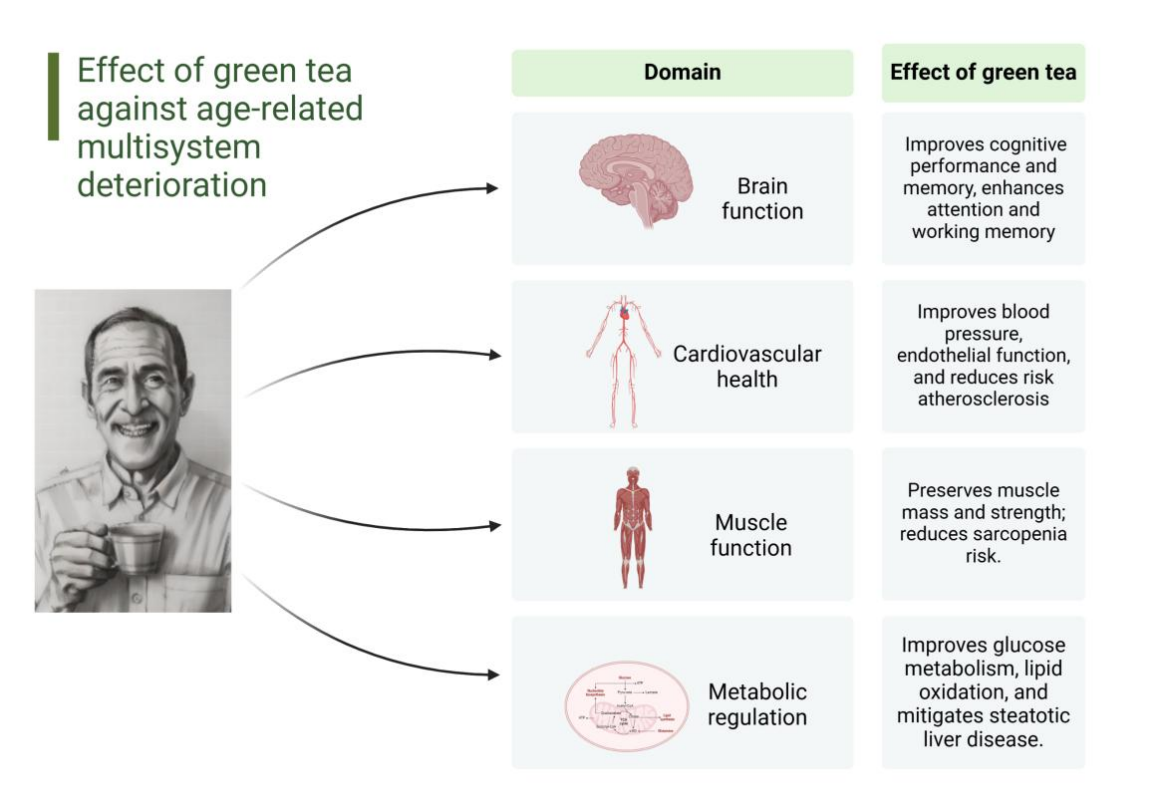
Summary of green tea’s effects against age-related multisystem deterioration (*Created with* Biorender.com).

## 5. Perspective and conclusions

While GT cannot reverse the inexorable process of senescence, it exhibits a remarkable capacity to mitigate the twelve traditional hallmarks of aging through diverse and complementary mechanisms. This mechanistic versatility at the molecular and cellular levels suggests that identifying pleiotropic compounds capable of simultaneously addressing the interconnected network of aging pathways may offer a promising avenue for future anti-aging interventions. In this context, GT could serve as a compelling proof-of-concept, demonstrating how a naturally occurring preparation with multiple bioactive components can influence the complex biology of aging more comprehensively than specifically designed pharmaceuticals. Notably, the effects of GT have been found to translate into tangible clinical outcomes across multiple physiological systems that functionally decline with aging (Fig. 2). Accordingly, human studies have consistently linked GT consumption to improved cognitive function, cardiovascular health, muscle preservation, and metabolic regulation. Importantly, the evidence surrounding the anti-aging effects of GT challenges our conventional biomedical paradigm, which commonly privileges novel, patentable molecules over traditional natural compounds. This bias may have led to an undervaluation of complex botanical preparations like GT, whose multiple bioactive components may work synergistically in ways that single-molecule drugs cannot replicate. Embracing such complexity could open new avenues for anti-aging research beyond the limitations of reductionist pharmacological approaches.

However, several limitations in the existing literature warrant consideration. First, substantial variability exists in the concentrations and dosages of GT and EGCG across published investigations, accompanied by diverse intervention durations. Furthermore, numerous studies investigating GT failed to quantify polyphenol content, and several human trials inadequately documented beverage preparation methods. These methodological inconsistencies significantly impede cross-study comparisons and hinder the establishment of reliable dose-response relationships. Second, the anti-aging potential of GT catechins in humans may be constrained by several pharmacokinetic limitations - including poor bioavailability, inadequate systemic absorption following oral administration, limited biodistribution, extensive first-pass metabolism, and low accumulation in target tissues [174]. Potential strategies to address this issue include the co-administration of catechins with other bioactive compounds, the implementation of nanostructure-based drug delivery systems, and strategic molecular modifications [174]. Third, when evaluating GT as a pleiotropic anti-aging intervention, safety considerations must be paramount. In this regard, regulatory bodies - including the European Food Safety Authority Panel on Food Additives and Nutrient Sources Added to Food (EFSA ANS) - have established safety thresholds recommending that GT extract consumption be limited to doses providing less than 800 mg/day of EGCG due to potential hepatotoxicity concerns [175]. Nevertheless, the EFSA ANS has concluded that catechins from traditionally prepared GT infusions and reconstituted beverages with comparable compositions generally demonstrate acceptable safety profiles under the presumption of safety approach, provided consumption is in accordance with documented intake levels across European Member States [175]. Another safety consideration warranting attention is the notable capacity of tea plants to accumulate aluminum in their foliar tissues at concentrations exceeding those of most other edible plants [176]. However, current evidence suggests that this characteristic poses negligible health risks to consumers when tea is consumed within conventional parameters [176]. Fourth, we acknowledge the risk of potential interactions between GT compounds and commonly prescribed medications in older adults. Accordingly, a recent review has documented that GT catechins can significantly impact drug pharmacokinetics, with 72% of analyses revealing decreased systemic exposure (18-99%) for various medications, including cardiovascular drugs, while one analysis noted increased exposure for sildenafil, and 22% showed no significant effects [177]. The primary mechanisms of GT-drug interactions appear to involve enhancement of P-glycoprotein efflux activity along with reduced drug solubility. Notably, case reports have linked these pharmacokinetic changes to alterations in drug efficacy and safety [177], underscoring their clinical importance and the need for further research in elderly populations. Lastly, human observational studies on GT and EGCG are potentially prone to reverse causation. This limitation arises from the possibility that healthier individuals may be more inclined to consume GT, thereby confounding the observed associations and making it challenging to establish a clear causal relationship.

It is also noteworthy that several critical research gaps remain to be addressed. Longitudinal studies using standardized GT preparations are necessary to establish optimal dosing regimens and determine whether benefits follow a hormetic response curve. Investigating potential synergies between GT and other anti-aging interventions - including exercise [178], caloric restriction [179], or pharmacological agents [180] - could reveal enhanced effects beyond what either approach achieves alone. Exploring genetic and epigenetic factors that influence individual responsiveness to GT could enable more personalized approaches to healthspan extension. Furthermore, developing advanced drug delivery systems for GT compounds could enhance their bioavailability and targeted effects [181], potentially leading to novel strategies for harnessing the full potential of GT in promoting healthy aging and longevity. Finally, further research is required to elucidate the effects of geographic origin, cultivation methods, and processing techniques on the concentration and bioavailability of bioactive compounds in GT preparations intended for human use. As our understanding of aging biology will continue to evolve in the future, it is anticipated that the study of GT may provide valuable insights into strategies for extending both lifespan and healthspan in a harmonious manner.
